# Hyperspectral Characteristic Band Selection and Estimation Content of Soil Petroleum Hydrocarbon Based on GARF-PLSR

**DOI:** 10.3390/jimaging9040087

**Published:** 2023-04-20

**Authors:** Pengfei Shi, Qigang Jiang, Zhilian Li

**Affiliations:** College of Geo-Exploration Science and Technology, Jilin University, Changchun 130026, China

**Keywords:** hyperspectral, characteristic band, soil petroleum hydrocarbon content, estimation, GARF-PLSR

## Abstract

With continuous improvements in oil production, the environmental problems caused by oil exploitation are becoming increasingly serious. Rapid and accurate estimation of soil petroleum hydrocarbon content is of great significance to the investigation and restoration of environments in oil-producing areas. In this study, the content of petroleum hydrocarbon and the hyperspectral data of soil samples collected from an oil-producing area were measured. For the hyperspectral data, spectral transforms, including continuum removal (CR), first- and second-order differential (CR-FD, CR-SD), and Napierian logarithm (CR-LN), were applied to eliminate background noise. At present, there are some shortcomings in the method of feature band selection, such as large quantity, time of calculation, and unclear importance of each feature band obtained. Meanwhile, redundant bands easily exist in the feature set, which seriously affects the accuracy of the inversion algorithm. In order to solve the above problems, a new method (GARF) for hyperspectral characteristic band selection was proposed. It combined the advantage that the grouping search algorithm can effectively reduce the calculation time with the advantage that the point-by-point search algorithm can determine the importance of each band, which provided a clearer direction for further spectroscopic research. The 17 selected bands were used as the input data of partial least squares regression (PLSR) and K-nearest neighbor (KNN) algorithms to estimate soil petroleum hydrocarbon content, and the leave-one-out method was used for cross-validation. The root mean squared error (RMSE) and coefficient of determination (R^2^) of the estimation result were 3.52 and 0.90, which implemented a high accuracy with only 8.37% of the entire bands. The results showed that compared with the traditional characteristic band selection methods, GARF can effectively reduce the redundant bands and screen out the optimal characteristic bands in the hyperspectral data of soil petroleum hydrocarbon with the method of importance assessment, which retained the physical meaning. It provided a new idea for the research of other substances in soil.

## 1. Introduction

Since the 20th century, with the rapid growth of the global economy, oil consumption has also increased. In oil exploitation, transportation, storage, production, and processing, accidental leakage and the unreasonable discharge of oily wastewater have caused serious pollution to soil [1]. Petroleum hydrocarbon is a type of persistent organic pollutant, and its low reactivity and antidegradation pose a serious threat to the ecological environment and human health [2,3]. To assess the potential risk of soil pollution and monitor oil pollution, researchers try to measure the content of petroleum hydrocarbons in soil. The common methods are gas chromatography, mass spectrometry, liquid chromatography, and solid-phase microextraction [4]. However, the above methods are complex, costly, time-consuming, and unsuitable for large-area detection. Therefore, a model for the rapid and accurate estimation of soil petroleum hydrocarbon content should be established.

With continuous advancements in remote sensing technology, hyperspectral imaging is developed on the basis of imaging spectroscopy. Hyperspectral imaging can record the reflectance information of targets through thousands of closely arranged wavelengths [5,6]. Compared with traditional optical remote sensing (single-band and multi-band) with a low spectral resolution, it has more imaging channels, continuous spectral range, and more flexible spectral selectivity, which greatly improve the detection capability of targets [7]. Hyperspectral imaging can also reduce the phenomena of different objects having the same spectrum and the same objects having different spectra and detect substances that cannot be detected using traditional remote sensing technology.

Valuable achievements have been attained using hyperspectral technology, such as the estimation of soil heavy metal content, and such technology has been applied to other related fields [8,9]. Considerable research has shown that hyperspectral imaging is closely related to the moisture [10,11,12,13], nutrient [14,15,16,17,18,19], and heavy metal contents [20,21,22,23,24,25,26] and other indicators [27,28] of soil. The application of hyperspectral imaging in the oil pollution detection of soil has also achieved accomplishments. For instance, Foudan et al. [29] utilized hyperspectral images to detect oil spill areas and showed that hyperspectral imaging can eliminate the limitations of traditional methods to the greatest extent. The distribution of oil spill areas by using hyperspectral imaging is also more accurate than that by using traditional methods. Horig et al. [30] used Hymap data to identify oil-contaminated soil and found that they are effective for detecting oil pollution in soil via visible-infrared spectroscopy. A spectral band of 1730 nm can be used as the key characteristic band for petroleum hydrocarbon detection. Kuhn et al. [31] proposed the concept of a hydrocarbon index for hyperspectral imaging and considered that the larger the hydrocarbon index is, the higher the degree of hydrocarbon accumulation will be. Fan et al. [32] measured the hyperspectral data and petroleum hydrocarbon content of samples and analyzed the relationship between soil spectral characteristics and petroleum hydrocarbon content by using a univariate prediction model and stepwise regression method. However, there are some shortcomings in the method of feature band selection, such as large quantity, time of calculation, and unclear importance of each feature band obtained. Meanwhile, redundant bands easily exist in the feature set, which seriously affects the accuracy of the inversion algorithm.

Feature engineering is the key to hyperspectral applications. Compared with the traditional hyperspectral feature extraction methods (PCA, LDA, ICA, etc.) [33], this article proposes a new characteristic band selection method for soil petroleum hydrocarbon hyperspectral imaging based on Genetic Algorithm and Random Forest (GARF). GARF regards the importance of hyperspectral bands as the evaluation index and selects a subset of hyperspectral images as characteristic bands, which can contain most of the important information with a small number of bands. It can effectively reduce the dimension of hyperspectral bands and make the characteristic subset have a clear physical meaning, which provides a basis for further study of the physicochemical mechanism in the hyperspectral imaging of soil petroleum hydrocarbon. The characteristic bands selected via GARF were used as input data of partial least squares regression (PLSR) and k-nearest neighbor (KNN) to estimate the content of petroleum hydrocarbons in soil samples. The experimental results showed that this method obtains an excellent estimation result, which provides a novel method for large-scale accurate, rapid, and low-cost detection of soil petroleum hydrocarbon. We conducted all experiments on a computer with Intel^®^ Core™ (Santa Clara, CA, USA) i7-8700K CPU at 3.70 GHz, 16 GB running memory, and all of our algorithms were coded using python and the open-source modules Scikit-learn.

## 2. Materials and Methods

### 2.1. Soil Sample Collection and Spectral Data Acquisition

**Figure 1:**
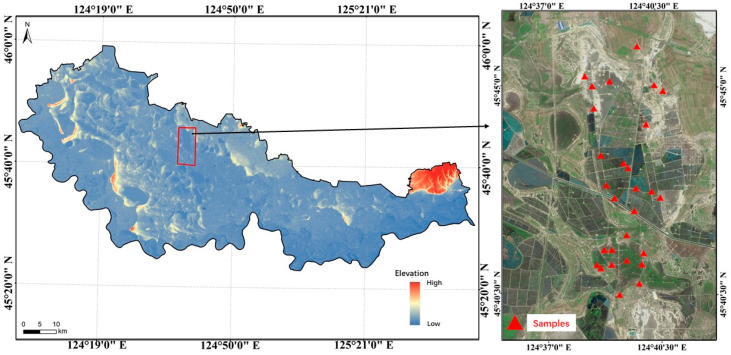
The position of sampling sites in the study area.

The soil sample collection area was in Daqing City, Heilongjiang Province, which is located in the southwest of Daqing Oilfield. The sampling area has a temperate continental climate, with four distinct seasons, annual precipitation about 600 mm, and abundant water resources. The types of soil in the sampling area are chernozem and meadow soil; the pH ranges from 5.4 to 10.7, with an average of 9.0, belonging to weak alkaline soil [34]. When collecting soil samples, we took each sampling point as the center with sampling area 1 m^2^. Five random locations of soil were stripped of impurities and collected at each sampling grid, and then were put into the sampling bag and mixed fully, with a sampling depth within 15 cm. In consideration of objective factors, such as oil well layout, oil leakage, and traffic, 28 soil samples were collected in the study area (Figure 1). The petroleum hydrocarbon was measured using the gas chromatography method (BS-EN-ISO 16703:2011). Three of the soil samples had abnormal values, in which the concentration of petroleum hydrocarbon was considerably higher than other samples. The analysis showed that the abnormal samples were caused by mixing black oily sludge. Oily sludge is a kind of sludge mixed with heavy oil, such as asphalt and various refined and residue oil, which is not inherent in nature. Therefore, the three abnormal samples were removed, and the spectral data of the remaining 25 soil samples were measured (Table 1). An ASD FieldSpec3 spectro-radiometer (Analytical Spectral Devices Inc., Boulder, CO, USA) was applied to measure the spectra of soil samples, and the spectral range was determined to be 350–2500 nm. A 50 W halogen lamp was used as the light source to simulate sunlight, and the zenith angle was 30°. The field of view angle and the measurement distance of the probe were 15° and 5 cm, respectively. A whiteboard was used for reflection calibration, and measurements of the soil spectrum were carried out in a dark laboratory. Each sample was measured 15 times to ensure the accuracy of the spectral data. The average spectral value was calculated as the reflectance value of the sample. In the ranges of 350–379 nm and 2401–2500 nm, the signal-to-noise ratio was low. Thus, the bands before 380 nm and after 2400 nm were discarded. The sampling interval of the spectrometer was 1 nm; hence, 2021 bands were obtained in a range of 380–2400 nm. Owing to the high spectral resolution, information overlap existed between adjacent bands, which made the results highly vulnerable to noise. Therefore, the spectral data were resampled, and the interval was 10 nm.

### 2.2. Spectral Data Preprocessing

**Figure 2:**
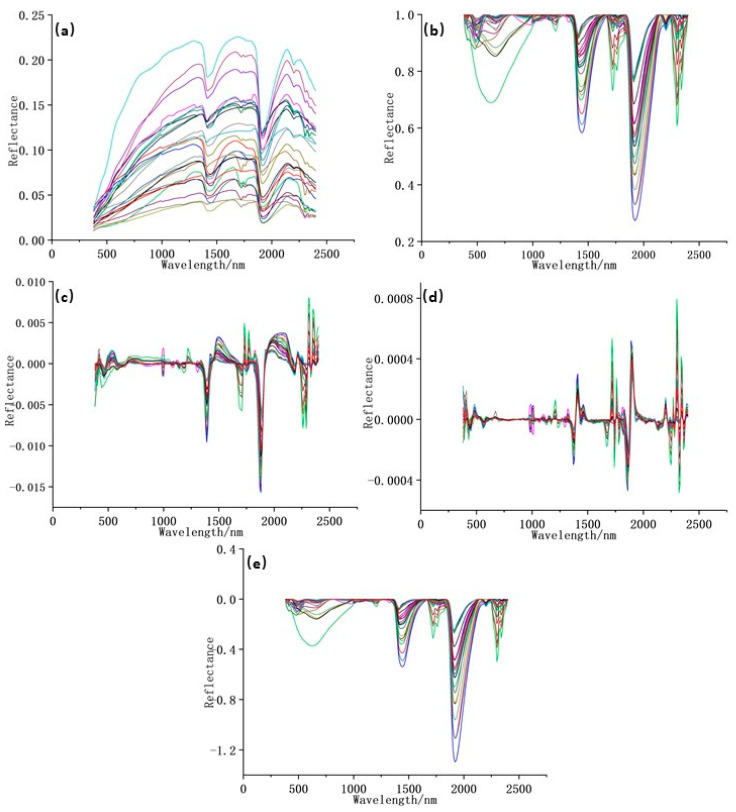
Five spectral forms of samples: (**a**) initial reflectance; (**b**) continuum removal of reflectance; (**c**) first derivative of reflectance; (**d**) second derivative of reflectance; (**e**) napierian logarithm of reflectance.

On the basis of denoising and resampling, the initial spectrum was processed through continuum removal (CR), which can highlight the absorption, reflection, and emission characteristics of the spectrum [35]. First- and second-order differential (CR-FD, CR-SD) and the Napierian logarithm (CR-LN) were used to eliminate the noise in the background of the CR spectrum [36]. The initial spectrum of soil samples is shown in Figure 2a. The initial reflectance of the soil samples was between 0 and 0.25, and the fluctuation shape of the spectral curves of each sample was similar. In the visible bands, with the increase in wavelengths, the reflectance gradually enlarged and tended to be stable at 1200 nm. In the near-infrared bands, the reflectance of the samples fluctuated greatly. Two distinct absorption valleys were distributed at approximately 1400 and 1900 nm, and a slightly sunken one existed at 2200 nm. Figure 2b–e show the results of four different transformations of the initial spectrum. All the four transformations can amplify the initial spectrum. After transformations, the reflectance fluctuated more remarkably at approximately 1400, 1900, and 2200 nm.

### 2.3. Model Principle

Feature selection is a technology for dimension reduction, which is the process of selecting a feature subset from a feature set. In the hyperspectral field, feature selection is also called band selection [37]. Compared with feature selection, feature extraction may obtain slightly better results in most cases, but the features converted through feature extraction have poor interpretability. The inherent physical meaning of hyperspectral imaging is also lost. However, feature selection directly selects a feature subset from the original feature set, such that it retains the physical meaning as opposed to feature extraction [38]. In view of the above reason, band selection is more widely used in hyperspectral dimensionality reduction than feature extraction. For hyperspectral imaging, dozens of optimal characteristic bands are difficult to select from hundreds or thousands of spectral bands on account of hundreds of millions of band combinations. At present, two strategies, namely group search and point-by-point search, are applied to select a feature subset from a complex feature set. The former method is to generate several candidate feature subsets continuously, reserve relatively good subsets, feed back to the subset generation strategy, evaluate and select new subsets, and repeat the above process until the candidate feature subsets meet the set requirements. The optimal feature subset can be considered as the result of feature selection. The latter method is to start from an empty set and select one (or more) feature from the feature set to join the subset until the subset contains the required number of features or to start from the set including all features and delete the features that meet the conditions one by one (or more) until the number of remaining features meets the set requirements.

#### 2.3.1. Genetic Algorithm

Genetic algorithm (GA) is a typical group search algorithm, which is based on natural selection and genetic theory. It combines the survival of the fittest rules in the process of biological evolution with the random information exchange mechanism of chromosomes in a population [39,40]. Furthermore, GA is also an efficient global optimization search algorithm, which abandons the traditional search strategy and simulates the biological evolution process in nature to search a feature subset randomly. It regards a possible solution to a problem as an individual or a chromosome of a population and codes each individual into a symbol string to simulate the evolution process of Darwinian genetic selection and natural elimination. In accordance with the evolutionary rules of survival of the fittest, each individual is evaluated using a predetermined objective fitness function, and the better population is retrieved continuously. At the same time, the global parallel search method is used to search the optimal individuals in the optimization population to reserve an optimal solution. The implementation process of GA is as follows:

1. Coding: The transformation of a feasible solution of a practical problem from its solution space to the search space that can be processed using GA is called coding. The most common coding method is binary coding.
2. Population analysis and design: GA randomly generates a certain number of individuals, from which better individuals are selected to form the initial population. In the iterative process, the larger the population size is, the higher the chance to obtain an optimal solution, and the smaller the possibility of the algorithm falling into a local minimum. However, the large population size will lead to an increase in the time consumption of the algorithm.
3. Fitness function: A fitness function is applied to evaluate the optimization process of individuals in the population and estimate the degree close to the optimal solution.
4. Crossover: GA imitates the process of gene recombination into new chromosomes in nature. Some genes in chromosomes are exchanged between two pairs of chromosomes, and a crossover operator is used to form two new individuals.
5. Mutation: Mutation is introduced to induce the formation of new individuals and increase the ability to find the optimal solution.
6. Termination of calculation: The individual with the maximum fitness value reserved in the evolution process is selected as the output of the optimal solution.

#### 2.3.2. Random Forest

Random Forest (RF) is an integrated algorithm composed of numerous decision trees. The idea of RF is to build an excellent tree, which needs to select excellent features. Therefore, the importance of each feature must be judged. RF randomly changes the value of a feature and then compares the error rate of the test set before and after the change. It adopts a point-by-point search strategy. The difference in error rate is regarded as the importance of the feature, and the mean decrease in accuracy (MDA) is considered the index to evaluate the feature importance of RF [41].

(1) $$MDA=\frac{1}{n}\sum_{t=1}^{n}\left(errOOB_{t}-errOOB^{\prime }{}_{t}\right)$$

where n is the number of based learners, and $errOOB^{\prime }{}_{t}$ is the out-of-pocket error after noise is added. The more the MDA index decreases, the greater the effect of the corresponding characteristic on the estimation results, and the higher its importance will be.

#### 2.3.3. Partial Least Squares Regression

Partial least squares regression (PLSR) is a multiple linear regression algorithm, which is one of the most used regression algorithms in hyperspectral imaging [42]. When hyperspectral data are used to estimate soil petroleum hydrocarbon content, the independent variable (*X*) in the estimation model is soil spectral data, and the dependent variable (*Y*) is the petroleum hydrocarbon content. The process of PLSR is summarized as follows:

(2) $$X=TP^{T}+E$$

(3) $$Y=UQ^{T}+F$$

where X is the predictive matrix, Y is the response matrix, and T and U are the projection matrices of X and Y, respectively. P and Q are the orthogonal load matrices, and matrices E and F are the error terms.

#### 2.3.4. K-Nearest Neighbor

K-nearest neighbor (KNN) is a supervised machine learning algorithm. The basic idea of KNN is to traverse the training set, find the k training samples closest to the new sample according to the distance formula, and use the majority voting principle to determine the prediction value of the new sample. It is widely used for dealing with classification and regression problems [43].

#### 2.3.5. Performance Evaluation Scales

To evaluate the performance of models in this study, root mean squared error (RMSE) and coefficient of determination ($R^{2}$) were used as indicators to assess the accuracy and stability of the models:

(4) $$R^{2}=1-\frac{\sum_{i=1}^{n}{\left(y_{i}-Y_{i}\right)}^{2}}{\sum_{i=1}^{n}{\left(y_{i}-\bar{y}\right)}^{2}}$$

(5) $$RMSE=\sqrt{\frac{1}{n}\sum_{i=1}^{n}{\left(y_{i}-Y_{i}\right)}^{2}}$$

where n is the number of samples, $y_{i}$ is the measured value, $Y_{i}$ is the predicted value, and $\bar{y}$ is the average of the measured values. The lower the RMSE is, the closer the $R^{2}$ is to 1, and the higher the accuracy and stability of the estimation model are.

## 3. Results and Discussion

**Figure 3:**
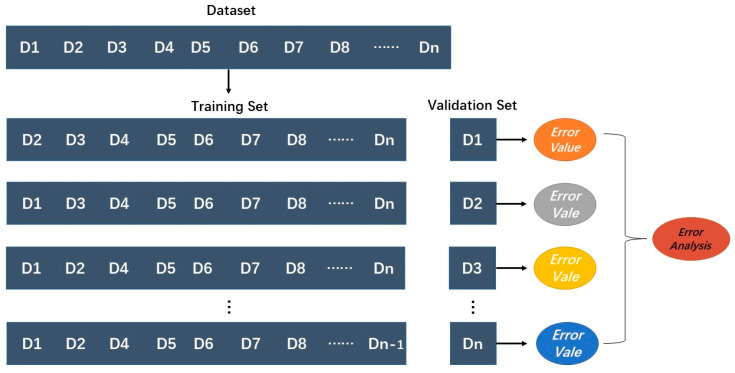
Schematic diagram of leave-one-out method.

We used GA to screen the hyperspectral imaging and select the characteristic bands of soil petroleum hydrocarbon. However, the combination of characteristic bands found using GA was still complex. To further eliminate redundant features, RF was used to sort the importance of characteristic bands and conducted secondary screening to select the optimal characteristic bands of soil petroleum hydrocarbon, whose importance was greater than the average. The optimal characteristic bands were regarded as the input data of the PLSR and KNN to estimate the soil petroleum hydrocarbon content. In order to facilitate the determination of parameters, we used the Grid Search method in this experiment. The Grid Search method only needs to input the parameter range, and it can automatically adjust parameters and output the optimal results, avoiding the tedious work of manual adjustment. It is a very suitable automatic parameter adjustment method for small data sets. The data set was divided into two parts by using the leave-one-out method. The advantage of this method is that every iteration used the maximum number of samples as the training set and made the estimation results clearly reflect the accuracy of the model (Figure 3).

### 3.1. Selection of Optimal Characteristic Bands

**Figure 4:**
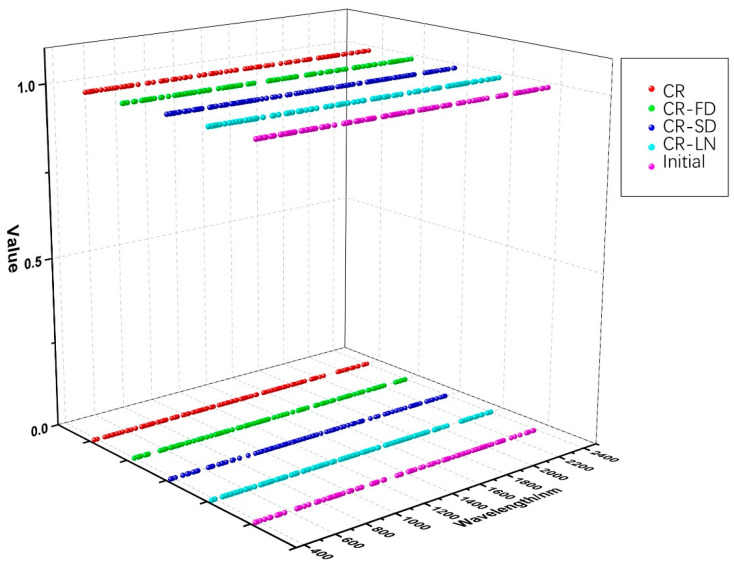
Distribution of bands marked using GA.

GA was used to screen the initial and transformation spectra in accordance with the genetic mechanism and natural selection. The population size, iterations, crossover rate, and mutation rate of GA were 50, 100, 0.6, and 0.02, respectively. Figure 4 shows the distribution of bands marked using GA for five types of spectra. On the basis of the operation of selection, crossover, and mutation with continuous genetic iteration, the bands with better fitness function values were reserved (bands were marked as 1), whereas the bands with worse fitness function values were eliminated (bands were marked as 0). The numbers of characteristic bands for initial, CR, CR-FD, CR-SD, and CR-LN spectra were 108, 91, 98, 108, and 91, respectively (Table 2). Characteristic bands of five spectral forms are listed in Appendix A.

**Figure 5:**
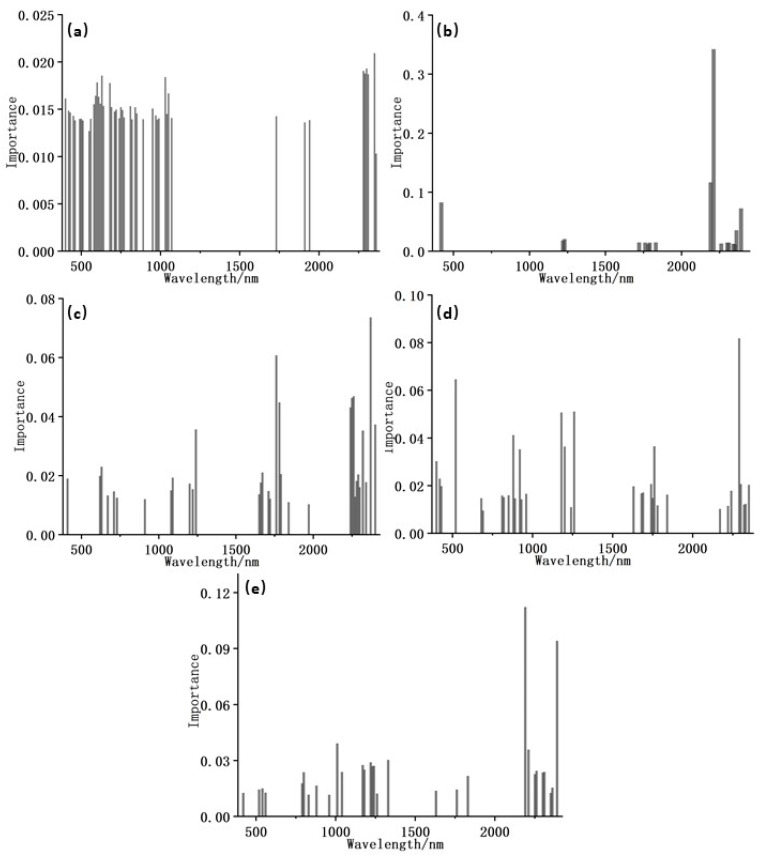
Distribution of optimal characteristic bands after RF selection: (**a**) Initial-GARF; (**b**) CR-GARF; (**c**) CR-FD-GARF; (**d**) CR-SD-GARF; (**e**) CR-LN-GARF.

Table 2 shows that the number of bands selected using GA was still large, and data redundancy remained among spectral bands. The band combination was screened for a second time through the function of out-of-bag estimation of RF, and the bands with greater-than-average importance were selected as the optimal characteristic bands. This process not only greatly reduced the dimension of the bands but also retained the most important characteristic information, which provided an effective data basis for the subsequent estimation of soil petroleum hydrocarbon content. Table 3 shows the number and importance of optimal characteristic bands after secondary screening by using RF. Figure 5 depicts the distribution of optimal characteristic bands and the importance value.

### 3.2. Estimation Accuracies of Soil Petroleum Hydrocarbon Content

PLSR is a nonparametric regression analysis method based on factor analysis, which is highly suitable for modeling under the condition of a small number of high-dimensional samples. Many studies have shown that it has excellent performance in the spectral estimation of soil material content [44,45,46]. Table 3 indicates that the optimal characteristic bands of soil petroleum hydrocarbon hyperspectral imaging selected using GARF can extract most of the important information in only a small amount of bands: CR-GARF had the best effect, in which 82% of the important information was contained in 17 characteristic bands (420, 1220, 1230, 1720, 1760, 1780, 1790, 1830, 2190, 2210, 2260, 2300, 2310, 2340, 2350, 2360, 2390 nm, only 8.37% of entire bands). Partial characteristic bands selected by GARF were similar to the conclusions reached by predecessors: Cloutis et al. studied the reflection characteristics of petroleum hydrocarbon in the visible near-infrared bands and summarized that there were two absorption bands near 1730 nm and 2310 nm [47]; Gao et al. perceived that in the near-infrared bands, crude oil solid had relatively obvious absorption bands near 1700 nm and 2300 nm [48]; nine absorption peaks of oil-contaminated soil were identified in the 1725–14,000 nm range through indoor spectral measurements by Zhu et al., with three of them (1725, 2310, 2348 nm) located in the visible near-infrared bands [49]; Feng et al. screened the above characteristic bands and believed that the double absorption peaks near 1748 nm and 2330 nm were the key wavebands for detecting the petroleum hydrocarbon in soil [50]; based on the sampling analysis of Gudong Oilfield, Fan et al. determined that the 1690–1790 nm range was the main area for estimating the content of petroleum hydrocarbon in soil [32]; Wang et al. conducted spectral analysis of soil samples with different oil contents using the visible near-infrared bands and found that the double absorption near 1748 nm and 2330 nm can be used as characteristic bands for soil oil pollution research [51]. The characteristic bands obtained in the above study obviously coincided with the characteristic bands selected in this article within band ranges of 1720–1790 nm and 2300–2350 nm, which proved the rationality of GARF.

**Figure 6:**
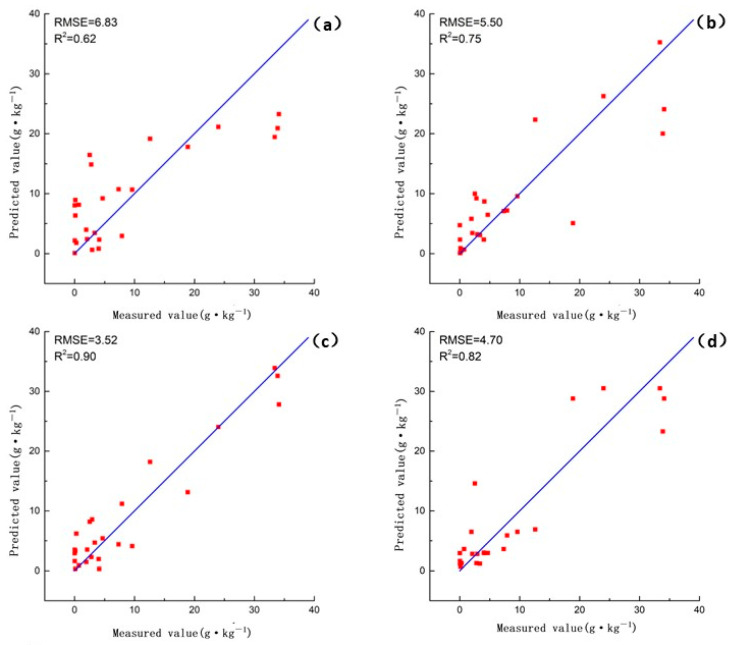
Scatter plots of the predicted versus measured soil petroleum hydrocarbon content: (**a**) Initial-PLSR; (**b**) CR-PLSR; (**c**) CR-GARF-PLSR; (**d**) CR-GARF-KNN.

**Figure 7:**
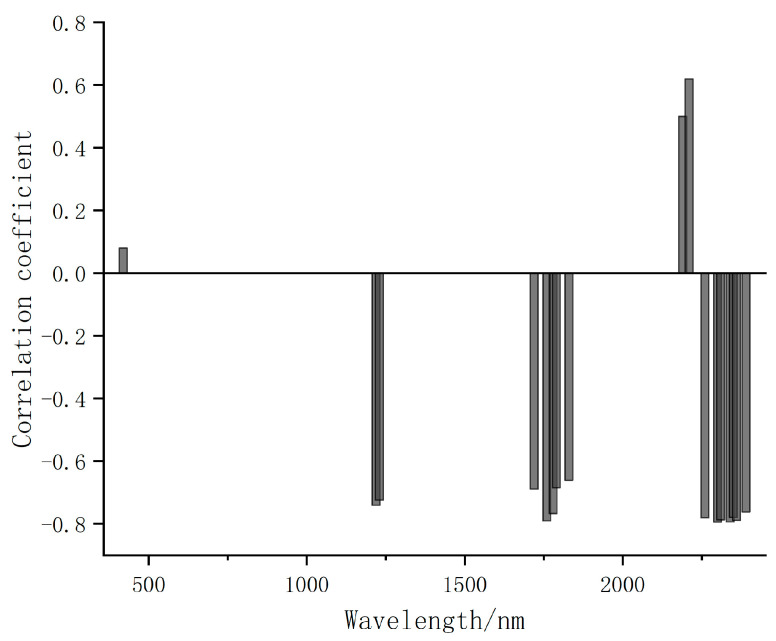
Correlation coefficient between soil petroleum hydrocarbon content and CR-GARF optimal characteristic bands.

**Figure 8:**
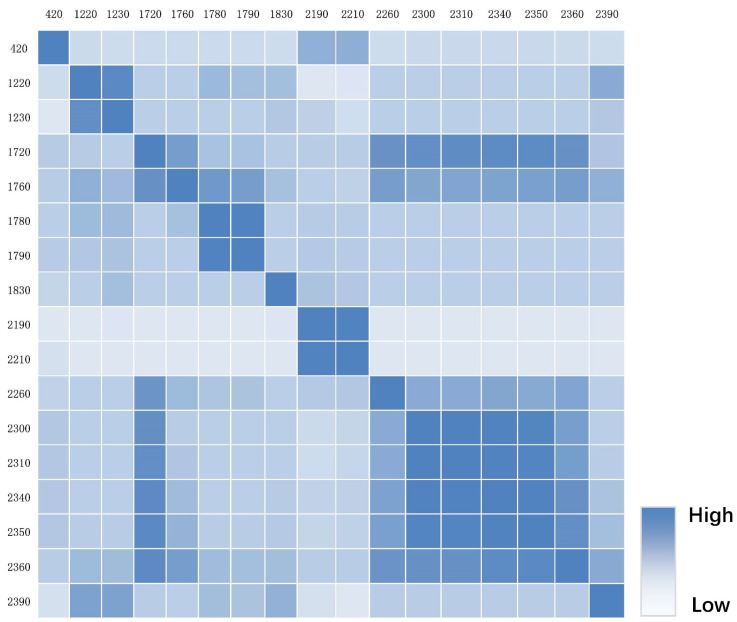
Heatmap of the correlation coefficient of CR-GARF optimal characteristic bands (wavelength/nm).

Then, we input the initial, CR, and CR-GARF spectra into PLSR and KNN for comparative analysis to validate the performance in estimation of soil petroleum hydrocarbon content. Table 4 and Figure 6 show that CR-GARF-PLSR can accurately estimate the content of soil petroleum hydrocarbon with fewer bands (RMSE = 3.52, $R^{2}$ = 0.90), which indicated that GARF can validly reduce redundant bands and screen out the optimal characteristic bands of soil petroleum hydrocarbon. Compared with the estimation accuracy of Initial-PLSR (RMSE = 6.83, $R^{2}$ = 0.62), that of CR-PLSR (RMSE = 5.50, $R^{2}$ = 0.75) was improved, which demonstrated that CR can highlight the characteristic information of the initial spectrum, remove background noise, and improve the estimation result. From Figure 6c,d, PLSR was more accurate than KNN in the estimation of soil petroleum hydrocarbon content. For validation of GARF in the band selection of the CR spectrum, the characteristic bands of the CR spectrum selected using GARF calculated the correlation coefficient among bands and between the petroleum hydrocarbon content of soil samples. Figure 7 demonstrated that the optimal characteristic bands selected using GARF had a high correlation with soil petroleum hydrocarbon content (|correlation coefficient| ≥ 0.6) [52]. The correlation coefficient among bands was insignificant except for adjacent intervals (Figure 8).

## 4. Conclusions

In this article, two tasks were implemented in order to estimate soil petroleum hydrocarbon content: a. laboratory analysis on the collected samples with an ASD spectro-radiometer; b. hyperspectral estimation based on laboratory analysis. For rapid and accurate estimation of soil petroleum hydrocarbon content, CR-GARF-PLSR was proposed. In the experiment, the RMSE and $R^{2}$ of the model were 3.52 and 0.90, which illustrated that it was an effective method in estimating soil petroleum hydrocarbon content. The experimental conclusions were as follows: 1. CR can eliminate the background noise in spectral data, highlight the absorption and reflection characteristics of spectral curves, and contribute to a more accurate estimation result. 2. GARF can effectively remove the redundant bands in soil petroleum hydrocarbon hyperspectral imaging and retain the optimal characteristic bands, which is a new method for feature selection based on machine learning. 3. Compared with other models, CR-GARF-PLSR had better performance in estimating soil petroleum hydrocarbon content, which provided a new idea for the research of other substances in soil. However, there are still some difficulties in the detection of oil pollution in soil by using hyperspectral technology. For instance, the differences in the composition of oil and the environment in different oil fields and the water content in crude oil will definitely have an impact on the measured spectra. The corresponding characteristic bands will also change accordingly, so the scalability and applicability of the model need further verification. At the same time, the method proposed in this paper still needs indoor testing, which will increase the cost of the experiment. In the near-infrared band, absorbance is also a common spectral measurement parameter, which may be helpful in improving experimental accuracy.

In this study, we established a new model for hyperspectral characteristic band selection and estimation of soil petroleum hydrocarbon content, which achieved an excellent performance. Furthermore, the research in this paper can be improved in the future. Due to the limitation of policy, we only collected a small number of samples. If the number of samples is enough, we can use more advanced regression methods, such as XGBoost and convolutional neural network, to make the final result more accurate. Similarly, we cannot remove samples containing other contaminations, except for black oily sludge, because of the limited number of samples. If we can fulfill this assumption, our experimental accuracy may be improved. With the resolution of the mixed pixel and the noise generation in field applications, the combination of this research and airborne hyperspectral technology has great potential in large-scale accurate and low-cost rapid detection of soil petroleum hydrocarbon.

## Figures and Tables

**Table 1:** Statistical description of the measured petroleum hydrocarbon content of soil samples (Min: minimum; max: maximum; ave: average; SD: standard deviation; CV: coefficient of variation).

No.	Min (g/kg^−1^)	Max (g/kg^−1^)	Ave (g/kg^−1^)	SD (g/kg^−1^)	CV
25	0.0081	34.1	8.46	11.02	130.23%

**Table 2:** Statistical table of bands selected using GA.

Spectral Form	Number of Resampled Bands	Number of Selected Bands
Initial	203	108
CR	203	91
CR-FD	203	98
CR-SD	203	108
CR-LN	203	91

**Table 3:** Importance of optimal characteristic bands after secondary screening by using RF.

Spectral Form	No.	Ave	Sum
Initial-GA	47	0.0093	0.721
CR-GA	17	0.0102	0.822
CR-FD-GA	33	0.0110	0.810
CR-SD-GA	34	0.0093	0.818
CR-LN-GA	30	0.0110	0.790

**Table 4:** Estimation accuracies of soil petroleum hydrocarbon content.

Model	RMSE	R^2^
Initial-PLSR	6.83	0.62
CR-PLSR	5.50	0.75
CR-GARF-PLSR	3.52	0.90
CR-GARF-KNN	4.70	0.82

## Data Availability

Not applicable.

## Appendix A

**Table A1:** Characteristic bands of Initial selected by GA.

Position of Characteristic Bands (nm)											
400	420	430	450	460	490	500	510	550	560	580	590
600	610	620	630	640	680	690	710	720	740	750	760
770	810	820	840	850	890	950	970	980	990	1030	1040
1050	1070	1100	1110	1120	1130	1140	1150	1210	1220	1230	1260
1270	1280	1290	1300	1310	1320	1330	1350	1380	1390	1400	1410
1460	1480	1490	1500	1520	1540	1550	1560	1580	1620	1630	1650
1660	1670	1730	1740	1760	1770	1810	1840	1860	1880	1890	1910
1940	2020	2040	2060	2070	2080	2150	2160	2170	2180	2190	2210
2220	2240	2250	2260	2280	2290	2300	2310	2350	2360	2390	2400

**Table A2:** Characteristic bands of CR selected by GA.

Position of Characteristic Bands (nm)											
390	410	420	430	440	460	490	520	540	560	580	590
620	640	650	670	680	690	730	790	800	830	880	890
900	920	960	970	980	1010	1040	1050	1080	1140	1170	1180
1220	1230	1240	1260	1290	1330	1360	1390	1400	1460	1480	1500
1510	1540	1550	1560	1570	1610	1630	1660	1720	1760	1780	1790
1830	1850	1890	1910	1920	1930	1940	2000	2010	2030	2040	2060
2070	2080	2090	2100	2110	2120	2130	2140	2150	2190	2210	2250
2260	2300	2310	2340	2350	2360	2390					

**Table A3:** Characteristic bands of CR-FD selected by GA.

Position of Characteristic Bands (nm)											
380	390	410	450	460	500	510	520	530	540	550	560
580	620	630	670	710	730	740	760	780	790	810	820
840	870	880	900	910	930	940	1000	1030	1050	1060	1080
1090	1100	1120	1140	1150	1200	1220	1240	1340	1350	1360	1380
1390	1410	1440	1460	1480	1490	1500	1520	1530	1540	1610	1630
1640	1650	1660	1670	1710	1720	1760	1780	1790	1830	1840	1850
1880	1900	1960	1970	1990	2000	2030	2060	2070	2080	2110	2120
2160	2190	2230	2240	2250	2260	2270	2280	2290	2300	2320	2340
2370	2400										

**Table A4:** Characteristic bands of CR-SD selected by GA.

Position of Characteristic Bands (nm)											
400	420	430	440	450	470	480	510	520	540	570	580
590	600	610	620	670	680	690	710	740	760	770	780
810	820	830	850	860	870	880	890	910	920	930	940
960	980	1010	1040	1080	1100	1130	1180	1200	1240	1260	1280
1310	1330	1350	1380	1400	1410	1430	1440	1450	1470	1490	1530
1550	1560	1580	1600	1610	1630	1640	1680	1690	1740	1750	1760
1770	1780	1800	1810	1820	1840	1860	1870	1880	1890	1910	1940
1950	1970	1980	2000	2020	2040	2050	2070	2080	2090	2170	2190
2200	2210	2220	2240	2260	2290	2300	2320	2330	2350	2390	2400

**Table A5:** Characteristic bands of CR-LN selected by GA.

Position of Characteristic Bands (nm)											
390	410	420	430	440	450	490	500	510	520	540	560
580	600	610	620	630	640	680	690	730	750	760	770
790	810	830	840	850	900	950	960	970	980	1010	1030
1040	1050	1060	1070	1080	1140	1180	1210	1220	1230	1240	1250
1280	1330	1360	1390	1410	1440	1480	1500	1510	1540	1560	1570
1610	1630	1660	1720	1760	1780	1830	1850	1890	1920	1930	1960
1970	2000	2030	2040	2050	2070	2080	2100	2110	2120	2130	2140
2150	2210	2250	2300	2310	2360	2390					
